# Optimal follow-up intervals for different stages of chronic kidney disease: a prospective observational study

**DOI:** 10.1007/s10157-018-01684-4

**Published:** 2019-01-28

**Authors:** Keita Hirano, Daiki Kobayashi, Naoto Kohtani, Yukari Uemura, Yasuo Ohashi, Yasuhiro Komatsu, Motoko Yanagita, Akira Hishida

**Affiliations:** 1 Department of Nephrology, Kyoto University Graduate School of Medicine, Shogoin-Kawahara-cho 54, Sakyo-ku, Kyoto, 606-8507 Japan; 2 Department of Nephrology, St. Luke’s International Hospital, Tokyo, Japan; 3 Division of General Internal Medicine, Department of Medicine, St. Luke’s International Hospital, Tokyo, Japan; 4 Biostatistics Group, Center for Product Evaluation, Pharmaceuticals and Medical Devices Agency, Tokyo, Japan; 5 Biostatistics Division, Central Coordinating Unit, Clinical Research Support Center, The University of Tokyo Hospital, Tokyo, Japan; 6 Department of Integrated Science and Engineering for Sustainable Society, Chuo University, Bunkyo-ku, Tokyo, Japan; 7 Department of Healthcare Quality and Safety, Graduate School of Medicine, Gunma University, Maebashi, Gunma Japan; 8 Yaizu City Hospital, Yaizu, Shizuoka Japan

**Keywords:** Testing interval, Chronic kidney disease, CKD-JAC, Cohort study

## Abstract

**Background:**

Chronic kidney disease (CKD) is a public health challenge; however, evidence-based, optimal follow-up intervals for patients with CKD have not been identified. This study aimed to identify appropriate follow-up intervals for different stages of CKD.

**Methods:**

We studied 2682 patients with CKD. The number of patients experiencing a 50% increase in creatinine and those reaching end-stage renal failure were examined on the basis of their CKD stage. The renal function testing interval was defined as the estimated time for 0.1% of the patients with CKD to have a composite renal outcome, after adjusting for clinical risk factors. Transitions from CKD stage-based subgroups were analyzed using parametric cumulative incidence models. Other sensitivity analyses involved estimation of the time to renal event occurrence for 1% of patients.

**Results:**

Of the 913 patients (34%) who had a composite renal event, 29 had stage 3A (10.5%), 151 had stage 3B (16.3%), 429 had stage 4 (41.0%), and 304 had stage 5 CKD (70.9%). The estimated renal function testing intervals for patients with CKD were 6.0 months for stage 3A, 3.4 months for stage 3B, 2.0 months for stage 4, and 1.2 months for stage 5.

**Conclusions:**

The optimal follow-up intervals were longer for patients with lower CKD stages. These estimates are longer than those recommended by the current guidelines and serve as a reference for nephrologists in selecting an appropriate follow-up interval for each patient.

**Trial registration:**

UMIN clinical trial registry number: UMIN000020038.

**Electronic supplementary material:**

The online version of this article (10.1007/s10157-018-01684-4) contains supplementary material, which is available to authorized users.

## Introduction

In 2002, the National Kidney Foundation of the United States proposed the concept of chronic kidney disease (CKD) with the objectives of comprehensively covering a wide range of kidney diseases, including various underlying diseases, and developing measures for continuous management of this chronic condition [1]. Recently, CKD has been recognized as a major global public health problem with increasing incidence and prevalence worldwide [2–6].

Nephrologists are responsible for performing periodic follow-up examinations to assess the therapeutic efficacy and revise patients’ therapeutic regimens according to disease progression [7]. The follow-up should be frequent to identify CKD progression, but excessive follow-up increases patients’ burden and medical costs. Following up with different patients at different intervals is considered effective because the rate of renal function decline varies widely from person to person. The international guidelines by Kidney Disease: Improving Global Outcomes (KDIGO) provide guidance on the frequency of renal function assessment according to disease staging based on the glomerular filtration rate (GFR), and the severity of CKD based on the presence of albuminuria [8]. Additionally, the Evidence-based Practice Guideline for the treatment of CKD of the Japanese Society of Nephrology, adapted from international guidelines, provides guidance for the frequency of follow-up by a nephrologist [7].

However, the intervals suggested in these guidelines were based only on a consensus among experts that renal function decreases more rapidly as CKD severity increases. These recommendations have not been confirmed by clinical or epidemiological studies [8]. On the basis of previous researches [10–12], we aimed to identify appropriate follow-up intervals for different stages of CKD by defining these optimal intervals according to the time to occurrence of a composite renal event in 0.1% of the patients in respective CKD subgroups.

## Materials and methods

### Data sets

Data from the Chronic Kidney Disease Japan Cohort (CKD-JAC) were analyzed in this study. This was a prospective, multicenter study conducted in Japan from September 2007 to March 2013, with enrollment taking place from September 2007 to December 2008. We initially recruited 3087 patients. Japanese patients with CKD stage 3 or greater at 17 medical institutions with a nephrologist on staff were followed up for up to 4 years. Baseline data for these patients have already been published in previous reports [14–17].

**Fig. 1:**
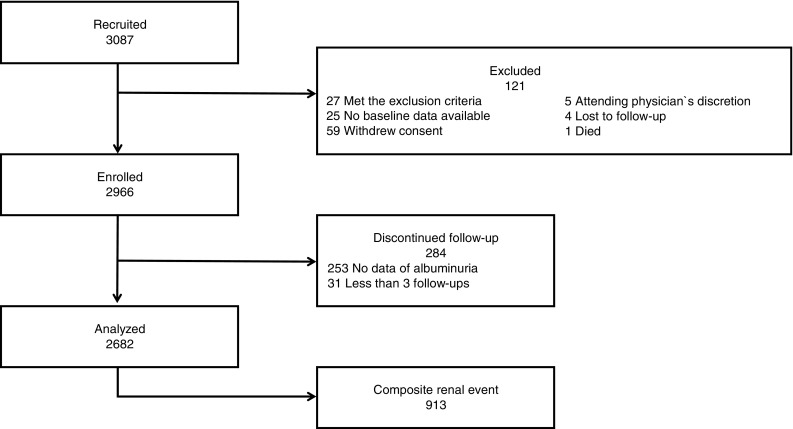
Patient disposition. Of the 3087 patients who enrolled in the CKD-JAC study, 121 were excluded, and a cohort of 2966 patients was analyzed. Composite renal outcomes consisted of either the start of treatment for end-stage renal failure (i.e., beginning dialysis or undergoing renal transplant) or a 50% increase in the baseline creatinine level, with time to the occurrence of the earlier of these events defined as the time to renal event occurrence. CKD-JAC, Chronic Kidney Disease Japan Cohort

To be eligible for the study, patients had to be either ethnic Japanese or Asian patients, aged between 20 and 75 years, residing in Japan, with an estimated glomerular filtration rate (eGFR) of 10–59 mL/min/1.73 m^2^ (or 10–49 mL/min/1.73 m^2^ for patients aged ≥ 65 years). Patients with polycystic kidney disease, HIV infection, hepatic cirrhosis, or a malignancy, transplant recipients, patients previously on chronic dialysis, and patients refusing to provide informed consent were all excluded from the study. Complete patient disposition is shown in Fig. 1.

The following formula of the Japanese Society of Nephrology for estimating serum creatinine was used to estimate the eGFR:

$${\text{eGFR }}={\text{ 194 }} \times {\text{ C}}{{\text{r}}^{ - {\text{1}}.0{\text{94}}}} \times {\text{ Ag}}{{\text{e}}^{ - 0.{\text{287}}}}\left( { \times 0.{\text{739 if female}}} \right).$$

The study protocol was approved by the ethics committee of each participating medical institution and was conducted in compliance with the principles of the Declaration of Helsinki. Informed consent was obtained from all participating patients (UMIN clinical trial registry number: UMIN000020038).

### Measurements

Patient data used in the study consisted of both central measurements and local measurements requested by investigators, but this information focused on locally determined serum creatinine values. The study data included the monthly measured serum creatinine levels obtained from the initial test date until censored. ‘Censored’ was defined as the start of end-stage renal failure treatment, which involved the start of hemodialysis, artificial dialysis, or renal transplant, refusal to continue study participation, transfer to another medical institution, or discontinuation or completion of observations for another reason.

Other baseline data included age at the start of examination, sex, CKD stage, presence of albuminuria, and underlying disease. Centrally determined albuminuria values were used. The CKD stages, based on calculated eGFR values (mL/min/1.73 m^2^), were stage 3A (45 to < 60), stage 3B (30 to < 45), stage 4 (15 to < 30), and stage 5 (< 15). The numbers of patients experiencing a 50% increase in the baseline creatinine level and those reaching end-stage renal failure were examined according to the CKD stage.

### Endpoints

Data from a previously studied large cohort indicated that a 40% reduction in the eGFR is consistent with a 50% increase in the baseline creatinine level [9, 18, 19]. Our study used renal event occurrence as a composite renal endpoint, defined as either 50% increase in the baseline creatinine level, or the start of treatment for end-stage renal failure (i.e., the start of dialysis or renal transplant), whichever occurred first. A 50% increase in the baseline creatinine level was determined after a 50% increase was observed in three consecutive serum creatinine tests, and the time of the first observed increase was used to determine the time to a 50% increase in the baseline creatinine level.

### Statistical analysis

Survival analyses were conducted to estimate the parametric cumulative incidence curve for the time to renal composite outcome, which was estimated from log-normal-regression models for the cumulative incidence function based on interval-censored data [21–24]. The log-normal model that best fit the data and had the smallest calculated Akaike information criterion value from among the log-normal model distribution, log-logistic model distribution, Weibull distribution, and exponential distribution was used to generate the cumulative incidence curve [25, 26]. With the study patients classified according to the CKD stage (3A, 3B, 4, and 5), the time to renal event occurrence for 0.1% of the patients was calculated from parametric cumulative incidence curves fit by CKD stage, and bootstrapping was used to calculate the 95% confidence intervals. To calculate the 95% confidence intervals, the lower bound 0.1% point was calculated 3000 times for bootstrap samples, obtained by sampling with the replacement of a number of patients equal to the number of patients in each subgroup, with the confidence intervals determined on the basis of the one-sided 2.5% points of the distributions. The lower bound 0.1% point was also estimated for the risk factors of age (< 65 or ≥ 65 years), sex, macroalbuminuria, and diabetes mellitus. Finally, 0.1% point and 95% confidence intervals were calculated for different combinations of these risk factors in the patients with CKD stage 5. For sensitivity analyses, 0.1% point was also estimated for different stages of CKD using a model adjusted for age and sex (Model 1) and a model adjusted for age, sex, albuminuria, and diabetes mellitus status (Model 2). Other sensitivity analyses estimated the time to renal event occurrence for the 1% threshold (Tables S1, S2).

SAS Release 9.4 (SAS Institute Inc., Cary, NC, USA) was used to conduct all statistical analyses.

## Results

### Baseline characteristics of the analyzed population

Baseline characteristics are shown in Table 1. The mean age of the 2682 patients was 60.5 years. Men outnumbered women (1657 men, 61.8%). The mean creatinine level of the overall population was 2.2 mg/dL, and 1014 patients (37.8%) had diabetes mellitus. During the observation period, 913 patients (34%) had a composite renal event, with an event observed in 29 patients with stage 3A CKD (10.5%), 151 with stage 3B CKD (16.3%), 429 with stage 4 CKD (41.0%), and 304 with stage 5 CKD (70.9%). Of the 913 patients with a compound renal event, 786 experienced a 50% increase in baseline creatinine levels, and 127 began treatment for end-stage renal failure.

**Table 1:** Patient characteristics

Patient characteristics	Total	CKD^a^ stage			
		3A	3B	4	5
	*n* = 2682	*n* = 277	*n* = 929	*n* = 1047	*n* = 429
Age ≧ 65 years	60.5 (11.5)	55.0 (13.2)	60.1 (11.8)	61.5 (10.6)	62.2 (10.6)
Male	1657 (61.8%)	171 (61.7%)	599 (64.5%)	638 (60.9%)	249 (58.0%)
Serum creatinine	2.2 (1.1)	1.1 (0.17)	1.4 (0.25)	2.3 (0.53)	4.0 (0.89)
Proteinuria	1604 (59.8%)	102 (36.8%)	455 (49.0%)	696 (66.5%)	351 (81.8%)
Diabetes mellitus	1014 (37.8%)	90 (32.5%)	342 (36.8%)	402 (38.4%)	180 (42.0%)
Hypertension	2207 (82.3%)	209 (75.5%)	735 (79.1%)	886 (84.6%)	377 (87.9%)
Composite renal event	913 (34.0%)	29 (10.5%)	151 (16.3%)	429 (41.0%)	304 (70.9%)
50% increase in creatinine	786 (29.3%)	28 (10.1%)	147 (15.8%)	408 (39.0%)	203 (47.3%)
ESRD^b^	127 (4.7%)	1 (0.4%)	4 (0.4%)	21 (2.0%)	101 (23.5%)

Data are presented as n (%) and mean (SD). Proteinuria and albuminuria were characterized by a relatively high rate of urinary excretion of albumin, typically greater than 300 mg per 24-h period. Diabetes mellitus was defined either by physician diagnosis or on the basis of the use of antidiabetic medication. Hypertension was defined either by physician diagnosis or on the basis of the use of antihypertensive medication

^a^Chronic kidney disease

^b^End-stage renal disease

### Estimation of follow-up intervals for evaluating renal function

**Fig. 2:**
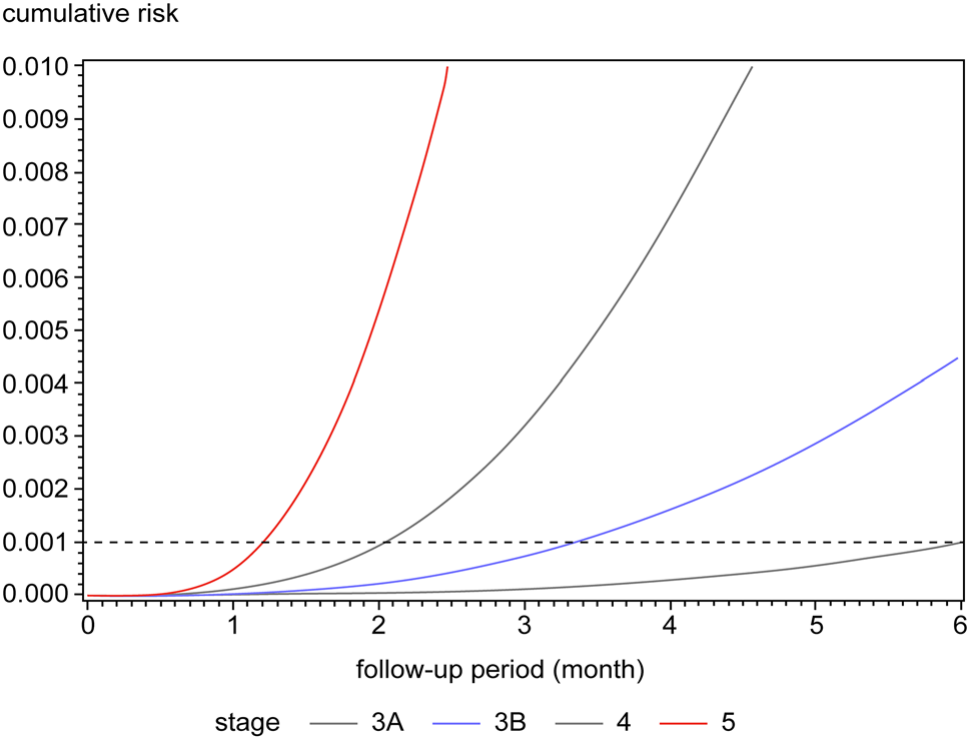
Unadjusted cumulative incidence of composite renal outcomes according to baseline CKD stage using parametric model. The proportion of patients with CKD who experienced a composite renal outcome is shown as a function of time. The cumulative incidence curves were estimated by means of parametric cumulative incidence models for interval-censored data. The dashed horizontal line marks the 0.1% threshold for the occurrence of the composite renal event. CKD, Chronic kidney disease

Cumulative incidence curves are shown by CKD stage, as determined by the eGFRs, in Fig. 2. The time to composite renal event occurrence in 0.1% of patients with CKD increased with decreasing baseline CKD stages. The estimates were 6.0 months for CKD stage 3A, 3.4 months for stage 3B, 2.0 months for stage 4, and 1.2 months for stage 5 (Table 2).

**Table 2:** Interval between baseline testing and composite renal outcome development in 0.1% of the patients with chronic kidney disease

Intervals	CKD^a^ stage			
	3A	3B	4	5
No. of months (95% CI)	6.0 (3.8–9.9)	3.4 (2.4–4.8)	2.0 (1.6–2.5)	1.2 (1.0–1.6)

Confidence interval was determined using a percentile bootstrap method based on 10,000 resamples

^a^Chronic kidney disease

Model 1 (adjusted for age and sex) produced estimates almost identical to those of the unadjusted model. The estimates produced with Model 2 (additionally adjusted for macroalbuminuria, diabetes mellitus, and hypertension) were 1–2 months longer. These estimates were quite similar to the corresponding estimates at the 0.1% threshold (Table S3).

The trends for the estimates for patients with CKD stage 5 alone were shorter for patients older than 65 years, patients who were male, and for patients who had macroalbuminuria, history of diabetes mellitus, or history of hypertension (Table 3). The estimated follow-up interval was the shortest for patients with macroalbuminuria at 1.2 months, followed by patients with microalbuminuria at 2.8 months, and patients without albuminuria at 8.6 months.

**Table 3:** Interval between baseline testing and composite renal outcome development in 0.1% of the patients with chronic kidney disease stage 5

Variables	Interval between baseline testing and development of composite renal outcome
	No. of months (95% CI)
Age	
≧65 years	1.2 (0.9–1.6)
<65 years	1.3 (0.9–1.9)
Sex	
Male	1.2 (0.9–1.6)
Female	1.7 (1.1–2.6)
Diabetes mellitus	
Yes	1.2 (0.8–1.6)
No	1.3 (0.9–1.9)
Hypertension	
Yes	1.2 (0.9–1.5)
No	2.4 (1.3–4.5)
Albuminuria	
Macro	1.2 (1.0–1.6)
Micro	2.8 (1.3–6.5)
No	8.6 (4.9–18.4)

Macroalbuminuria is characterized by a relatively high rate of urinary excretion of albumin, typically 300 mg or more per 24-h period. Microalbuminuria is characterized by a relatively low rate of urinary excretion of albumin, typically between 30 and 300 mg per 24-h period. No albuminuria is characterized by a relatively high rate of urinary excretion of albumin, typically less than 30 mg per 24-h period

**Fig. 3:**
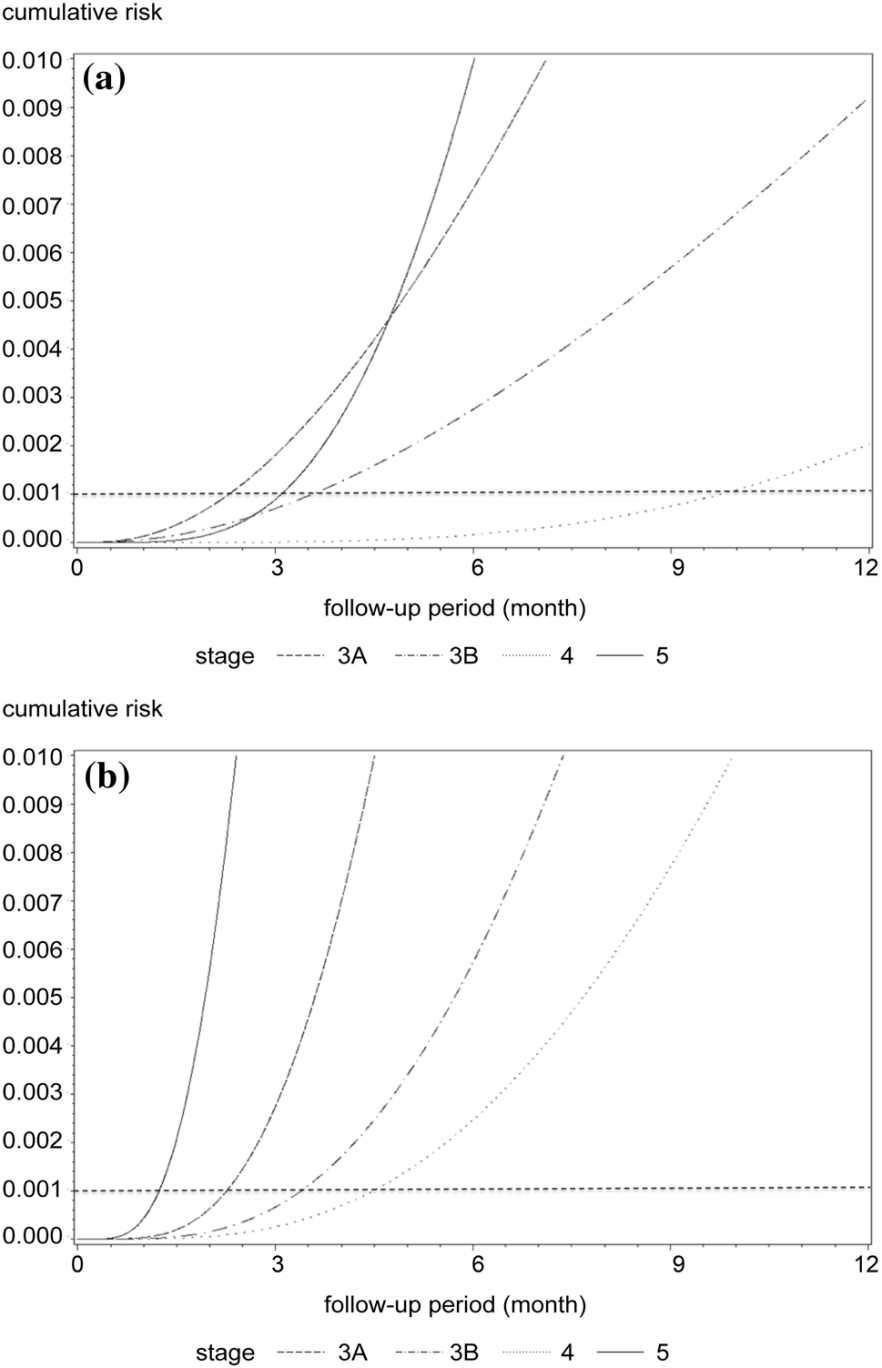
Unadjusted cumulative incidence of composite renal outcomes according to baseline CKD stage and proteinuria grades. The proportion of patients with CKD who experienced a composite renal outcome is shown as a function of time. The cumulative incidence curves were estimated by means of parametric cumulative incidence models for interval-censored data. The dashed horizontal line marks the 0.1% threshold for the occurrence of the composite renal event. *CKD* Chronic kidney disease

On the basis of the level of albuminuria and renal function CKD stage, a two-dimensional heat map was created. Cumulative incidence curves are shown in Fig. 3a, b. The level of albuminuria was classified as either severe (> 300 mg/g Cr) or not severe. Compared with the interval when the degree of proteinuria was not of concern, the difference was clear for up to 4.5 months when albuminuria was severe in stage 3A CKD and for 9.8 months when albuminuria was not severe (Table 4).

**Table 4:** Heat map of the frequency of monitoring by glomerular filtration rate and level of albuminuria

CKD^a^ stage	Albuminuria	
	Not severe	Severe
	No. of months (95% CI)	
3A	9.8 (5.4–19.9)	4.5 (2.4–8.9)
3B	3.6 (1.5–8.8)	3.4 (2.3–4.9)
4	2.3 (0.8–6.9)	2.3 (1.9–2.7)
5	3.1 (1.5–6.7)	1.2 (1.0–1.6)

^a^Chronic kidney disease

Redefining the optimal follow-up interval as the time of composite renal endpoint occurrence in 1% of the patients with CKD increased the time estimates by a factor of 2–2.5, relative to the estimates calculated for occurrence in 0.1% of the population (Tables S2, S3).

### Estimation of follow-up intervals for evaluating renal and cardiovascular disease events

We analyzed the number and percentage of subjects who developed cardiovascular disease (CVD) events by stage and urine albumin level, which revealed that CVD events increased as the CKD stage and proteinuria increased. The CVD event almost always preceded a renal composite outcome. However, in patients with high CKD stages and proteinuria, a renal composite outcome preceded CVD (Table S4). We redefined a renal composite event along with a CVD event as an outcome. The estimated renal function testing intervals were 0.96 [95% confidential intervals (CI) (0.19, 4.6)] months for CKD 3A, 2.0 [95% CI (1.3, 3.1)] months for stage 3B, 1.5 [95% CI (1.1, 2.0)] months for stage 4, and 0.90 [95% CI (0.65, 1.2)] months for stage 5 (Table S5).

## Discussion

Our findings suggest that renal function at the initial examination is the most relevant factor for determining the interval for additional renal function assessments. During the observation period, a composite renal event occurred in approximately 10% of the patients with an initial CKD stage 3A, 20% of those with stage 3B, 40% of those with stage 4A, and 70% of those with stage 5. Thus, the event occurrence increased with increasing CKD stage. The optimal intervals for follow-up examinations of renal function that we calculated were 6.0 months for CKD stage 3A, 3.4 months for stage 3B, 2.0 months for stage 4, and 1.2 months for stage 5. Thus, the time interval decreased as the stage increased, indicating the need for more frequent follow-up for patients with a higher CKD stage. Interestingly, the findings also indicated that intervals longer than those recommended in the current guidelines are sufficient for lower CKD stages.

The Evidence-based Practice Guideline for the Treatment of CKD of the Japanese Society of Nephrology also recommends follow-up intervals for renal function assessments [7]. Based on the expert opinion, factors from the findings of cohort research in the general Japanese population were adapted for the recommendations in the 2012 KDIGO guidelines to suit the Japanese patients [8]. According to proteinuria severity, the guidelines recommend that intervals of 3–6 months for CKD stage 3A, 3 months for stage 3B, 1–3 months for stage 4, and 1 month for stage 5 should be implemented. These intervals are very close to the intervals that we calculated for each subgroup in our study.

Men are thought to experience a more rapid decrease in the renal function than women [27, 28]. In our study, the follow-up interval for CKD stage 5 was 1.2 months for men, compared to 1.7 months’ follow-up interval for women. Furthermore, the follow-up intervals for patients with CKD stage 5 were 1.2 months if macroalbuminuria was present, 2.8 months if microalbuminuria was present, and 8.6 months if albuminuria was absent. It is significant that these differences according to the albuminuria status are more pronounced compared to those recommended in the current guidelines. In addition, the results of the heat map created in our study showed that in stage 3A CKD, when proteinuria is not severe, long screening intervals are acceptable.

As patients with CKD have various underlying diseases, practicing nephrologists select follow-up intervals using their expertise and patients’ age, sex, renal function, concurrent diseases, and other relevant factors. Although selecting follow-up intervals according to clinician expertise and CKD guideline recommendations is essential for preventing CKD progression, routine care should ideally be determined by the time to composite renal event occurrence.

Recent healthcare marketing efforts, advocacy, and trust in public health authorities have resulted in recommendations for excessive laboratory testing; however, an excessive testing can adversely affect the prognosis of other chronic diseases [30–32]. Nephrologists must understand the importance of developing rational screening programs based on the best available evidence. Our estimated intervals are universally longer than those recommended by the Japanese CKD guidelines, indicating that longer follow-up intervals are satisfactory, particularly for patients with lower CKD stages (i.e., stages 3A/3B) and those with stage 5 CKD with microalbuminuria or no proteinuria. Our study determined that longer follow-up intervals than those recommended in the current guidelines are often acceptable.

A previous report has documented that cardiovascular events occur more frequently than renal events, especially in the early stages of CKD [33]. Our study revealed that overall shortening of intervals especially that of the CKD stage 3A had occurred. This unexpected result was caused by the fact that some patients developed a CVD event early after observation.

The present study has a few limitations. The first limitation is the generalizability of results. The follow-up intervals in this study were based only on the incidence of a composite renal event adjusted for CKD risk factors. We did not factor in the potential benefits and risks or cost-effectiveness of periodic renal function testing, although the cost-effectiveness of CKD screening is an important method for increasing awareness [34, 35]. Although specific treatments administered are expected to influence the rate of renal function decline, we did not collect sufficient information on treatments to factor this into the analyses. It would have been possible to consider individual treatments administered to patients and effects of treatment differences. Many of the CKD-JAC patients receive advanced treatment (e.g., antihypertensives) by a nephrologist. Therefore, caution is needed when generalizing the study findings to all Japanese patients with CKD.

The second limitation is the overly small sample sizes in the subgroups; because of these small sample sizes, we did not classify patients according to CKD heat maps by combination of three proteinuria groups, four GFR grades and underlying diseases. Given the diverse range of diseases underlying CKD, an appropriate classification of these diseases must be considered; a larger sample size is needed for detailed analyses based on the underlying disease type.

The third limitation is the optimal interval which should be calculated using tools directly related to those evaluations [10]. Thus, CVD events should ideally be evaluated using echocardiography or electrocardiography.

The final limitation is the use of a lower bound 0.1% point as the threshold for selecting optimal intervals. Obviously, our intervals would have been longer had we used a threshold of 1% (Tables S1, S2). However, the sensitivity analysis of a previous study [10] led us to select intervals that were consistent with those used in clinical practice and are generally acceptable. Our 1.2-month interval for high-risk patients with CKD stage 5 is indeed close to that used in clinical practice. The threshold used for estimating evaluation intervals of osteoporosis in older women had a 10% lower bound [10], but we decided that a 0.1% threshold would better suit our patients because the composite renal event that we used was more conservative, and the signs of progression should be identified as early as possible. The interval we recommend is appropriate to prevent the worsening of CKD; in clinical practice, studies to address the optimal frequency of delivering adequate care and patient education are needed. Frequent visits may increase medical and resource expenditure, but they may promote intensive patient education and reassurance.

A major strength of this study was the use of a large data pool from approximately 3000 patients in a multicenter cohort, which is one of the largest cohorts of patients with CKD in Japan.

## Conclusions

The optimal follow-up intervals are longer for patients with lower CKD stages. These estimates are longer than those recommended by the relevant guidelines, and they serve as a reference for nephrologists to select appropriate follow-up intervals for their patients.

## Electronic supplementary material

Below is the link to the electronic supplementary material.

Supplementary material 1 (DOCX 19 KB)

Supplementary material 2 (DOCX 19 KB)

Supplementary material 3 (DOCX 19 KB)

Supplementary material 4 (DOCX 78 KB)

Supplementary material 5 (DOCX 65 KB)

## References

[1] Jha V, Garcia-Garcia G, Iseki K, Li Z, Naicker S, Plattner B, Saran R, Wang AY, Yang CW (2013). Chronic kidney disease: global dimension and perspectives. Lancet.

[2] Go AS, Chertow GM, Fan D, McCulloch CE, Hsu CY (2004). Chronic kidney disease and the risks of death, cardiovascular events, and hospitalization. N Engl J Med.

[3] Weiner DE, Tighiouart H, Amin MG, Stark PC, MacLeod B, Griffith JL, Salem DN, Levey AS, Sarnak MJ (2004). Chronic kidney disease as a risk factor for cardiovascular disease and all-cause mortality: a pooled analysis of community-based studies. J Am Soc Nephrol.

[4] Levey AS, Coresh J, Balk E, Kausz AT, Levin A, Steffes MW, Hogg RJ, Perrone RD, Lau J, Eknoyan G (2003). National Kidney Foundation. National Kidney Foundation practice guidelines for chronic kidney disease: evaluation, classification, and stratification. Ann Intern Med.

[5] Levey AS, Atkins R, Coresh J, Cohen EP, Collins AJ, Eckardt KU, Nahas ME, Jaber BL, Jadoul M, Levin A, Powe NR, Rossert J, Wheeler DC, Lameire N, Eknoyan G (2007). Chronic kidney disease as a global public health problem: approaches and initiatives—a position statement from kidney disease improving global outcomes. Kidney Int.

[6] Coresh J, Turin TC, Matsushita K, Sang Y, Ballew SH, Appel LJ, Arima H, Chadban SJ, Cirillo M, Djurdjev O, Green JA, Heine GH, Inker LA, Irie F, Ishani A, Ix JH, Kovesdy CP, Marks A, Ohkubo T, Shalev V, Shankar A, Wen CP, de Jong PE, Iseki K, Stengel B, Gansevoort RT, Levey AS (2014). Decline in estimated glomerular filtration rate and subsequent risk of end-stage renal disease and mortality. JAMA.

[7] Japan nephrology society (2012). [Special issue: Clinical practice guidebook for diagnosis and treatment of chronic kidney disease 2012]. Nihon Jinzo Gakkai Shi.

[8] Chapter 2: Definition, identification, and prediction of CKD progression. Kidney Int Suppl (2011). 2013;3:63–72. 10.1038/kisup.2012.65 PMC4089637 25018976

[9] Levey AS, Inker LA, Matsushita K, Greene T, Willis K, Lewis E, de Zeeuw D, Cheung AK, Coresh J (2014). GFR decline as an end point for clinical trials in CKD: a scientific workshop sponsored by the National Kidney Foundation and the US Food and Drug Administration. Am J Kidney Dis.

[10] Gourlay ML, Fine JP, Preisser JS, May RC, Li C, Lui LY, Ransohoff DF, Cauley JA, Ensrud KE, Study of Osteoporotic Fractures Research Group (2012). Bone-density testing interval and transition to osteoporosis in older women. N Engl J Med.

[11] Qaseem A, Hopkins RH, Sweet DE, Starkey M, Shekelle P (2013). Screening, monitoring, and treatment of stage 1 to 3 chronic kidney disease: a clinical practice guideline from the American College of Physicians. Ann Intern Med.

[12] Gourlay ML, Overman RA, Ensrud KE (2015). Bone density screening and re-screening in postmenopausal women and older men. Curr Osteoporos Rep.

[13] Imai E, Matsuo S, Makino H, Watanabe T, Akizawa T, Nitta K, Iimuro S, Ohashi Y, Hishida A, CKD-JAC Study Group (2008). Chronic kidney disease Japan cohort (CKD-JAC) study: design and methods. Hypertens Res.

[14] Iimuro S, Imai E, Watanabe T, Nitta K, Akizawa T, Matsuo S, Makino H, Ohashi Y, Hishida A (2014). Hyperbaric area index calculated from ABPM elucidates the condition of CKD patients: the CKD-JAC study. Clin Exp Nephrol.

[15] Akizawa T, Makino H, Matsuo S, Watanabe T, Imai E, Nitta K, Ohashi Y, Hishida A, Chronic Kidney Disease Japan Cohort Study Group (2011). Management of anemia in chronic kidney disease patients: baseline findings from Chronic Kidney Disease Japan Cohort Study. Clin Exp Nephrol.

[16] Iimuro S, Imai E, Watanabe T, Nitta K, Akizawa T, Matsuo S, Makino H, Ohashi Y, Hishida A, Chronic Kidney Disease Japan Cohort Study Group (2013). Clinical correlates of ambulatory BP monitoring among patients with CKD. Clin J Am Soc Nephrol.

[17] Imai E, Matsuo S, Makino H, Watanabe T, Akizawa T, Nitta K, Iimuro S, Ohashi Y, Hishida A (2010). Chronic Kidney Disease Japan Cohort study: baseline characteristics and factors associated with causative diseases and renal function. Clin Exp Nephrol.

[18] Stevens LA, Greene T, Levey AS (2006). Surrogate end points for clinical trials of kidney disease progression. Clin J Am Soc Nephrol.

[19] Greene T, Teng CC, Inker LA, Redd A, Ying J, Woodward M, Coresh J, Levey AS (2014). Utility and validity of estimated GFR-based surrogate time-to-event end points in CKD: a simulation study. Am J Kidney Dis.

[20] Lee M, Fine JP (2011). Inference for cumulative incidence quantiles via parametric and nonparametric approaches. Stat Med.

[21] Jeong JH, Fine J (2006). Direct parametric inference for the cumulative incidence function. J R Stat Soc.

[22] Jeong JH, Fine JP (2007). Parametric regression on cumulative incidence function. Biostatistics.

[23] Lindsey JC, Ryan LM (1998). Tutorial in biostatistics methods for interval-censored data. Stat Med.

[24] Klein JP, Moeschberger ML (2005). Survival analysis: techniques for censored and truncated data.

[25] Bozdogan H (1987). Model selection and Akaike’s information criterion (AIC): the general theory and its analytical extensions. Psychometrika.

[26] Posada D, Buckley T (2004). Model selection and model averaging in phylogenetics: advantages of Akaike information criterion and bayesian approaches over likelihood ratio tests. Syst Biol.

[27] Imai E, Horio M, Yamagata K, Iseki K, Hara S, Ura N, Kiyohara Y, Makino H, Hishida A, Matsuo S (2008). Slower decline of glomerular filtration rate in the Japanese general population: a longitudinal 10-year follow-up study. Hypertens Res.

[28] Hemmelgarn BR, Zhang J, Manns BJ, Tonelli M, Larsen E, Ghali WA, Southern DA, McLaughlin K, Mortis G, Culleton BF (2006). Progression of kidney dysfunction in the community-dwelling elderly. Kidney Int.

[29] Esserman L, Shieh Y, Thompson I (2009). Rethinking screening for breast cancer and prostate cancer. JAMA.

[30] Sawaya GF (2009). Cervical-cancer screening–new guidelines and the balance between benefits and harms. New Engl J Med.

[31] Partridge AH, Winer EP (2009). On mammography–more agreement than disagreement. New Engl J Med.

[32] Schwartz LM, Woloshin S, Fowler FJ, Welch HG (2004). Enthusiasm for cancer screening in the United States. JAMA.

[33] Nakayama M, Sato T, Miyazaki M, Matsushima M, Sato H, Taguma Y, Ito S (2011). Increased risk of cardiovascular events and mortality among non-diabetic chronic kidney disease patients with hypertensive nephropathy: the Gonryo study. Hypertens Res.

[34] Yarnoff BO, Hoerger TJ, Simpson SK, Leib A, Burrows NR, Shrestha SS, Pavkov ME (2017). The cost-effectiveness of using chronic kidney disease risk scores to screen for early-stage chronic kidney disease. BMC Nephrol.

[35] Komenda P, Ferguson TW, Macdonald K, Rigatto C, Koolage C, Sood MM, Tangri N (2014). Cost-effectiveness of primary screening for CKD: a systematic review. Am J Kidney Dis.

