# Supplementary material for: Optimal follow-up intervals for different stages of chronic kidney disease: a prospective observational study
Source: Clin Exp Nephrol. 2019 Jan 28;23(5):613–20. doi: 10.1007/s10157-018-01684-4 (PMC6469834; doi:10.1007/s10157-018-01684-4)
Supplement: Supplementary file 1 — Supplementary material 1 (DOCX 19 KB) [file 10157_2018_1684_MOESM1_ESM.docx]

**Optimal follow-up intervals for different stages of chronic kidney disease: A prospective observational study**

Clinical and Experimental Nephrology

Keita Hirano, Daiki Kobayashi, Naoto Kohtani, Yukari Uemura, Yasuo Ohashi, Yasuhiro Komatsu, Motoko Yanagita, and Akira Hishida.

**Corresponding author**

Keita Hirano, Department of Nephrology, Kyoto University Graduate School of Medicine, Shogoin-Kawahara-cho 54, Sakyo-ku, Kyoto 606-8507, Japan. E-mail: keita@kuhp.kyoto-u.ac.jp, Tel: +81-75-751-3860, Fax: +81-75-751-3859

**Table S1. Interval between baseline testing and composite renal outcome development in 1% patients with chronic kidney disease**

| CKD^a^ stage | Interval between baseline testing and development of renal outcome | | |
| --- | --- | --- | --- |
|  | Unadjusted | Adjusted (Model 1) | Adjusted (Model 2) |
|  | No. of months (95% CI) | | |
| 3A | 13.6 (10.1-19.3) | 15.0 (11.1-22.6) | 15.4 (12.0-26.7) |
| 3B | 8.5 (6.7-10.7) | 8.7 (6.9-11.0) | 10.1 (8.1-13.1) |
| 4 | 4.6 (3.8-5.4) | 4.7 (4.0-5.6) | 6.5 (5.3-7.9) |
| 5 | 2.5 (2.1-3.0) | 2.7 (2.2-3.2) | 3.7 (3.2-4.6) |

^a^*Chronic kidney disease*

*Model 1: Data were adjusted for age and sex.*

*Model 2: Data were adjusted for age, sex, proteinuria, diabetes, and hypertension.*
