# Supplementary material for: Optimal follow-up intervals for different stages of chronic kidney disease: a prospective observational study
Source: Clin Exp Nephrol. 2019 Jan 28;23(5):613–20. doi: 10.1007/s10157-018-01684-4 (PMC6469834; doi:10.1007/s10157-018-01684-4)
Supplement: Supplementary file 2 — Supplementary material 2 (DOCX 19 KB) [file 10157_2018_1684_MOESM2_ESM.docx]

**Optimal follow-up intervals for different stages of chronic kidney disease: A prospective observational study**

Clinical and Experimental Nephrology

Keita Hirano, Daiki Kobayashi, Naoto Kohtani, Yukari Uemura, Yasuo Ohashi, Yasuhiro Komatsu, Motoko Yanagita, and Akira Hishida.

**Corresponding author**

Keita Hirano, Department of Nephrology, Kyoto University Graduate School of Medicine, Shogoin-Kawahara-cho 54, Sakyo-ku, Kyoto 606-8507, Japan. E-mail: keita@kuhp.kyoto-u.ac.jp, Tel: +81-75-751-3860, Fax: +81-75-751-3859

**Table S2. Intervals for time to composite renal outcome in 0.1% and 1% of the patients**

| Percent transitioning to event | Intervals between baseline testing and development of composite renal outcome |
| --- | --- |
|  |  |
|  |  |
|  | No. of months (95% CI) |
| 0.10% | 1.5 (1.3-1.8) |
| 1% | 3.9 (3.5-4.4) |
