# Supplementary material for: Optimal follow-up intervals for different stages of chronic kidney disease: a prospective observational study
Source: Clin Exp Nephrol. 2019 Jan 28;23(5):613–20. doi: 10.1007/s10157-018-01684-4 (PMC6469834; doi:10.1007/s10157-018-01684-4)
Supplement: Supplementary file 3 — Supplementary material 3 (DOCX 19 KB) [file 10157_2018_1684_MOESM3_ESM.docx]

**Optimal follow-up intervals for different stages of chronic kidney disease: A prospective observational study**

Clinical and Experimental Nephrology

Keita Hirano, Daiki Kobayashi, Naoto Kohtani, Yukari Uemura, Yasuo Ohashi, Yasuhiro Komatsu, Motoko Yanagita, and Akira Hishida.

**Corresponding author**

Keita Hirano, Department of Nephrology, Kyoto University Graduate School of Medicine, Shogoin-Kawahara-cho 54, Sakyo-ku, Kyoto 606-8507, Japan. E-mail: keita@kuhp.kyoto-u.ac.jp, Tel: +81-75-751-3860, Fax: +81-75-751-3859

**Table S3. Interval between baseline testing and composite renal outcome development in 0.1% patients in different stages of chronic kidney disease**

| CKD^a^ stage | Interval between baseline testing and development of composite renal outcome | | |
| --- | --- | --- | --- |
|  | Unadjusted | Adjusted (Model 1) | Adjusted (Model 2) |
|  | No. of months (95% CI) | | |
| 3A | 6.0 (3.8-9.9) | 6.7 (4.2-12.0) | 7.4 (5.3-15.7) |
| 3B | 3.4 (2.4-4.8) | 3.5 (2.5-5.0) | 4.4 (3.1-6.4) |
| 4 | 2.0 (1.6-2.5) | 2.1 (1.7-2.7) | 3.1 (2.4-4.1) |
| 5 | 1.2 (1.0-1.6) | 1.3 (1.1-1.7) | 2.0 (1.6-2.5) |

^a^*Chronic kidney disease*

*Model 1: Data were adjusted for age and sex.*

*Model 2: Data were adjusted for age, sex, proteinuria, diabetes, and hypertension*
