# Supplementary material for: Optimal follow-up intervals for different stages of chronic kidney disease: a prospective observational study
Source: Clin Exp Nephrol. 2019 Jan 28;23(5):613–20. doi: 10.1007/s10157-018-01684-4 (PMC6469834; doi:10.1007/s10157-018-01684-4)
Supplement: Supplementary file 4 — Supplementary material 4 (DOCX 78 KB) [file 10157_2018_1684_MOESM4_ESM.docx]

**Optimal follow-up intervals for different stages of chronic kidney disease: A prospective observational study**

Clinical and Experimental Nephrology

Keita Hirano, Daiki Kobayashi, Naoto Kohtani, Yukari Uemura, Yasuo Ohashi, Yasuhiro Komatsu, Motoko Yanagita, and Akira Hishida.

**Corresponding author**

Keita Hirano, Department of Nephrology, Kyoto University Graduate School of Medicine, Shogoin-Kawahara-cho 54, Sakyo-ku, Kyoto 606-8507, Japan. E-mail: keita@kuhp.kyoto-u.ac.jp, Tel: +81-75-751-3860, Fax: +81-75-751-3859

**Table S4. Number of subjects and proportion who developed CVD events by stage and albumin urine classification**

| CKD^a^ stage | Number of subjects who developed renal outcome added CVD events  (Number of subjects who developed cardiac events prior to renal composite outcome) | | | |
| --- | --- | --- | --- | --- |
|  | Albuminuria | | | |
|  | Not severe | | Severe | |
|  | *n* | Proportion, % | *n* | Proportion, % |
| 3A | 10(9) | 5.7(5.1) | 3(3) | 2.9(2.9) |
| 3B | 31(29) | 6.5(6.1) | 33(24) | 7.3(5.3) |
| 4 | 23(22) | 6.6(6.3) | 60(39) | 8.6(5.6) |
| 5 | 5(4) | 6.4(5.1) | 30(21) | 8.5(6.0) |

^a^*Chronic kidney disease*
