# Supplementary material for: Optimal follow-up intervals for different stages of chronic kidney disease: a prospective observational study
Source: Clin Exp Nephrol. 2019 Jan 28;23(5):613–20. doi: 10.1007/s10157-018-01684-4 (PMC6469834; doi:10.1007/s10157-018-01684-4)
Supplement: Supplementary file 5 — Supplementary material 5 (DOCX 65 KB) [file 10157_2018_1684_MOESM5_ESM.docx]

**Optimal follow-up intervals for different stages of chronic kidney disease: A prospective observational study**

Clinical and Experimental Nephrology

Keita Hirano, Daiki Kobayashi, Naoto Kohtani, Yukari Uemura, Yasuo Ohashi, Yasuhiro Komatsu, Motoko Yanagita, and Akira Hishida.

**Corresponding author**

Keita Hirano, Department of Nephrology, Kyoto University Graduate School of Medicine, Shogoin-Kawahara-cho 54, Sakyo-ku, Kyoto 606-8507, Japan. E-mail: keita@kuhp.kyoto-u.ac.jp, Tel: +81-75-751-3860, Fax: +81-75-751-3859

**Table S5. Interval between baseline testing and composite renal outcome added CVD event development in 0.1% of the patients with chronic kidney disease**

| Intervals | CKD^a^ stage | | | |
| --- | --- | --- | --- | --- |
|  | 3A | 3B | 4 | 5 |
| No. of months (95% CI) | 0.96 (0.19-4.6) | 2.0 (1.3-3.1) | 1.5 (1.1-2.0) | 0.90 (0.65-1.2) |

^a^*Chronic kidney disease*
